# Detection of Common Deletional Alpha-Thalassemia Spectrum by Molecular Technique in Kelantan, Northeastern Malaysia

**DOI:** 10.5402/2012/462969

**Published:** 2012-07-19

**Authors:** B. Rosnah, H. Rosline, A. Wan Zaidah, M. N. Noor Haslina, R. Marini, M. Y. Shafini, F. A. Nurul Ain

**Affiliations:** Hematology Department, Hospital Universiti Sains Malaysia, Kelantan 16150 Kubang Kerian, Malaysia

## Abstract

Thalassemia is a hereditary blood disorder that results from genetic defects causing deficient synthesis of hemoglobin polypeptide chains. Although thalassemia mostly affects developing countries, there is limited knowledge of its accurate frequency and distribution in these regions. Knowing the prevalence of thalassemia and the frequency of responsible mutations is therefore an important step in the prevention and control program as well as treatment strategies. This study was performed to determine the prevalence and to study the spectrum of gene deletions that are responsible in **α**-thalassemia in Kelantan, located in northeastern Malaysia. A total 400 first-time blood donors from multiple areas of donation centre were chosen randomly. The presence of three types of **α**-thalassemia gene deletion in southeast Asian population which were -^SEA^deletion, -**α** ^3.7^ rightward deletion, and -**α** ^4.2^ leftward deletion was detected by using multiplex PCR method. 37 (9.25%) of blood donors were confirmed to have **α**-thalassemia deletion types. 34 (8%) were heterozygous for **α**3.7 deletion, 1 (0.25%) was heterozygous for **α**4.2 deletion, and 2 (0.5%) were heterozygous for SEA type deletion. Alpha-thalassemia-2 with 3.7 deletion was the most common determinant detected in Kelantan Malay compared to other ethnic groups. It has been noted that alpha-thalassemia-2 with 3.7 deletion is the most common type of **α**-thalassemia throughout the world.

## 1. Introduction

Thalassemia is a hereditary blood disorder that results from genetic defects causing deficient synthesis of hemoglobin polypeptide chains. It occurs with considerable frequency in ethnic groups tracing their origins to countries that border the Mediterranean Sea, the Middle East, and southeast Asia [1, 2].

In *α*-thalassemia, it is characterized by reduction or absence of the *α*-globin chains due to deletion or mutation of *α*-globin genes. The phenotype of individuals with deletional *α*-thalassemia is diverse and depends on the number of genes that have been deleted or mutated, as well as the type of deletion and mutation [3].

Normal individuals have 2*α* genes on each chromosome 16 (*αα*/*αα*) and they are located on the short arm [4]. The loss of one of two linked *α* genes in one chromosome is called alpha-thalassemia-2 (-*α*/*αα*). The two common varieties of this deletion are *α* ^3.7^ and *α* ^4.2^ types. Larger deletions are associated with the deletion of both *α* genes and are called alpha-thalassemia-1 (- -/*αα*). In southeast Asian the commonest type of alpha-thalassemia-1 is alpha-thalassemia-1 with SEA deletion [5]. Hb H disease reflects deletion of three out of four *α* genes (- -/-*α*). In Hb Barts fetalis syndrome, there are no functional *α* genes (- -/- -) [3].

It has been noted that alpha-thalassemia-2 is the most common type of *α* thalassemia throughout the world with estimated frequency of 30% to 50% [6].

Having heterozygous state of alpha-thalassemia-2 is not necessarily associated with hematological and clinical abnormality. In some heterozygous alpha-thalassemia-1 or homozygous alpha-thalassemia-2, the affected person may have mild microcytic and hypochromic RBC features. However, they are silent clinically. The problem arises when it interacts with other thalassemia genes or diseases since it will alter the disease phenotype and become clinically important. HbH disease is an example more severe form of *α* thalassemia resulting from the interaction between alpha-thalassemia-1 and alpha-thalassemia-2 [5, 7].

Although thalassemia mostly affects developing countries, there is limited knowledge of its accurate frequency and distribution in these regions. Knowing the prevalence of thalassemia and the frequency of responsible mutations is therefore an important step in the prevention and control program as well as treatment strategies [5, 8]. This study was performed to determine the prevalence and to study the spectrum of gene deletion that responsible in *α*-thalassemia in Kelantan, located in northeastern Malaysia.

## 2. Methodology

This study was carried out in the Hematology Department, Hospital Universiti Sains Malaysia. After written consent was obtained, 5 mls of blood from 400 first time blood donors were taken and collected into EDTA containers.

### 2.1. DNA Extraction

Genomic DNA was extracted and purified from whole blood sample using the Gentra Puregene Blood Kit (Qiagen, Germany). The concentration of DNA was determined by NanoDrop Spectrophotometer ND-1000 (NanoDrop Technologies, USA). To perform PCR for the deletion detection in this study, a concentration of DNA around 200 ng/*μ*L was required. Sample with the higher concentration of the DNA must be diluted to 200 ng/*μ*L prior to use in PCR.

### 2.2. Molecular Analysis for *α*-Thalassemia Detection

#### 2.2.1. Primer Dilution

All six sets of primer from manufacturer was provided in 100 *μ*M solution. The primers were diluted for PCR amplification used. 5 *μ*L of 100 *μ*M stock primer was diluted with 45 *μ*L of ddH20 in order to prepare 50 *μ*L of 10 *μ*M primer. The diluted primer was then shaking for 3 hours at room temperature.

#### 2.2.2. Detection of Common Deletion of *α*-Thalassemia Gene

DNA was tested for the presence of three types of *α*-thalassemia gene deletion in southeast Asian population which was -^SEA^ deletion, -*α* ^3.7^ rightward deletion and -*α* ^4.2^ leftward deletion. Presence of the normal *a2 globin gene* was confirmed either heterozygous or homozygous state of carrier.* LIS1 *gene 3′ UTR fragment which serves as a separate internal control was used for general amplification success. The protocol for PCR amplification was performed according to the multiplex PCR method described by Arnold S.-C. Tan et al. [9] with changes. The PCR products were run on a 1% agarose gel using 1X TBE buffer and lastly, the gel was visualized under ultraviolet (UV) light and the image was captured using AphaImager electronic imaging system (Alpha Innotech, USA). The image was archived in the computer for documentation.

## 3. Results

Among the 400 first-time blood donors studied, 37 of them (9.25%) were confirmed to have *α*-thalassemia of deletion type. 34 subjects (8%) were heterozygous for *α* ^3.7^ deletion while 1 subject (0.25%) was revealed to have heterozygous *α* ^4.2^ deletion, and 2 subjects (0.5%) had heterozygous SEA-type deletion.

We detected 10.5% of Kelantan Malay subjects and 2.4% of Kelantan Chinese subjects to have *α* ^3.7^ deletion. The *α* ^4.2^ deletion was detected only in Kelantan Chinese subjects. SEA deletion was detected in 1.2% of Kelantan Chinese subjects and only 0.3% of Kelantan Malay subjects. The 3 determinants were not detected in Kelantan Indian subjects. The results were summarized in Table 1.

## 4. Discussion

This study was performed to determine the prevalence and to study the spectra of gene deletions that are responsible in *α*-thalassemia in our region. Kelantan is situated in the northeast of Peninsular Malaysia, and majority of the population is Malay [10].

Samples were taken from blood donors during mobile blood donation. Since the mobile sessions were conducted in many parts of Kelantan, the samples were thus representing the true population of Kelantan.

In this study, we observed that the prevalence of *α*-thalassemia carrier with deletion type was 9.25%. *α*-thalassemia carrier with 3.7 deletion was the most common and occurred in high frequency in Kelantan Malay compared to other ethnic groups.

Similar observation was noted in Kuala Lumpur population by Othman A in 1994. He revealed that *α*-thalassemia 2 had two types, the commonest being deletion on 3.7 kb of DNA and the less common type involved deletion of 4.2 kb of DNA [11].

In 2005, Wee et al. revealed 15.8% of pregnant women attended one of the government hospitals in Kuala Lumpur were confirmed to have *α* thalassemia carrier. Out of this, 62% had 3.7 deletion, 3.8% had 4.2 deletion, and 24% had SEA deletion [12].

However, in their study, they noted that *α* thalassemia carrier with 3.7 deletion was distributed evenly among the 3 ethnic groups and the *α*-thalassemia carrier with 4.2 deletion was only seen in Malay. Similar observation for the SEA deletion in which it was seen in higher frequency among the Chinese [12].

The distributions of some determinants among the 3 ethnic groups from previous studies were not well correlated with the current study. This was mainly due to the difference in the ethnic distribution in the 2 areas studied. In this study, the determinants of detection were performed randomly regardless of the RBC parameters whereas in previous study, the PCR was only performed in samples with low MCV and MCH values. This kind of sample selection obviously increased the frequency of thalassemia carrier detection [13].

Our results from this study were comparable with other studies from various areas in southeast Asia. The prevalence of *α* thalassemia 2, particularly with 3.7 deletion was significantly higher compared to 4.2 deletion as well as to alpha-thalassemia-1 with SEA deletion. Northern Thailand is a place which has the highest frequency of alpha-thalassemia in the world. One study revealed that incidence of *α* thalassemia with 3.7 deletion was 16% in heterozygous state and 1.2% in homozygous state. For heterozygous SEA deletion, the incidence was about 14% [14]. One study revealed that the incidence of alpha-thal 2 and alpha-thal 1 traits were 12.0% and 4.3%, respectively, in southern Thailand [15].

In Singapore, a study revealed that in 990 young male adolescents studied, about 5% of hemoglobinopathies were detected. Out of this, alpha-thalassemia trait was the commonest and it occurred predominantly in the Chinese [16].

Similar observation was also seen in, Georgia, Iran and Brazil indicating that *α* thalassemia carrier with 3.7 deletion was much more common compared to other deletion determinants [6, 8, 17]. Thus, the finding of this study suggested that similar spectrum distribution of *α*-thalassemia deletion was not limited to southeast Asia countries but it seemed to occur worldwide.

## Figures and Tables

**Table 1:** Overall result of the 3 determinants among 3 ethnics in Kelantan.

Ethnic group	*α*-globin genotypes			Total
	- -*α* ^3.7^/*αα*	- -*α* ^4.2^/*αα*	- -^SEA^/*αα*
Malay (*n* = 315)	32	0	1	33
Chinese (*n* = 73)	2	1	1	4
Indian (*n* = 12)	0	0	0	0

Total (*n* = 400)	34	1	2	37
